# Supplementary material for: Fibroblastic reticular cells direct the initiation of T cell responses via CD44
Source: Nature. 2026 Jan 21;651(8106):752–62. doi: 10.1038/s41586-025-09988-8 (PMC12999478; doi:10.1038/s41586-025-09988-8)
Supplement: Supplementary file 1 — Reporting Summary [file 41586_2025_9988_MOESM1_ESM.pdf]

nature portfolio

Corresponding author(s): M. A. Degli-Esposti

Last updated by author(s): November 21, 2025

Reporting Summary

Nature Portfolio wishes to improve the reproducibility of the work that we publish. This form provides structure for consistency and transparency in reporting. For further information on Nature Portfolio policies, see our Editorial Policies and the Editorial Policy Checklist.

Statistics

For all statistical analyses, confirm that the following items are present in the figure legend, table legend, main text, or Methods section.

n/a	Confirmed	
☐	☒	The exact sample size (*n*) for each experimental group/condition, given as a discrete number and unit of measurement
☐	☒	A statement on whether measurements were taken from distinct samples or whether the same sample was measured repeatedly
☐	☒	The statistical test(s) used AND whether they are one- or two-sided<br>*Only common tests should be described solely by name; describe more complex techniques in the Methods section.*
☒	☐	A description of all covariates tested
☐	☒	A description of any assumptions or corrections, such as tests of normality and adjustment for multiple comparisons
☐	☒	A full description of the statistical parameters including central tendency (e.g. means) or other basic estimates (e.g. regression coefficient) AND variation (e.g. standard deviation) or associated estimates of uncertainty (e.g. confidence intervals)
☐	☒	For null hypothesis testing, the test statistic (e.g. *F*, *t*, *r*) with confidence intervals, effect sizes, degrees of freedom and *P* value noted<br>*Give P values as exact values whenever suitable.*
☒	☐	For Bayesian analysis, information on the choice of priors and Markov chain Monte Carlo settings
☒	☐	For hierarchical and complex designs, identification of the appropriate level for tests and full reporting of outcomes
☒	☐	Estimates of effect sizes (e.g. Cohen's *d*, Pearson's *r*), indicating how they were calculated

Our web collection on statistics for biologists contains articles on many of the points above.

Software and code

Policy information about availability of computer code

Data collection	BIAcore 3000 system (GE Healthcare)<br>Quantum-315 CCD detector at the MX2 beamline of the Australian Synchrotron<br>FACSymphony A3 analyser running FACSDiva software v9.1 (BD Biosciences)<br>Olympus BX60 epifluorescence microscope and Olympus DP-controller 3.1.1.267 (Olympus)<br>DMi8 Inverted Microscope (Leica Microsystems) with Leica Application Suite 2.7.3.9723<br>Leica SP5 and SP8 (Leica Microsystems) with Leica Application Suite v2.7.3.9723<br>Nikon AX R Ti2-E confocal microscope (Nikon) with NIS Elements v5.42.01<br>Zeiss LSM 980 with Airyscan Confocal Microscope (ZEISS) with Zen3.9<br>Amnis INSPIRE ImageStreamX (Cytek Biosciences)<br>Opera Phenix Plus High-Content Screening System (PerkinElmer)
Data analysis	Macromolecular Analysis:<br>CCP4 suite of programs v7.0.023<br>Scrubber2.0 (BioLogic Software, Campbell, ACT, Australia)<br>Coot v0.8.7<br>Phaser v2.7.17<br>Pymol v2.1.1<br>Buster Release 20180515<br><br>Flow Cytometry:<br>FlowJo Version v10 (BD Biosciences)

Amnis imageStreamX:
Image Data Exploration and Analysis Software (IDEAS) Version 6.2.187.0
CellProfiler Version 4.2.6

Imaging:
Fiji ImageJ2, Version 2.14. or 1.54f
Imaris, Version 10.2.0

Data Analysis and Visualisation:
GraphPad Prism, Version 10.6

For manuscripts utilizing custom algorithms or software that are central to the research but not yet described in published literature, software must be made available to editors and reviewers. We strongly encourage code deposition in a community repository (e.g. GitHub). See the Nature Portfolio guidelines for submitting code & software for further information.

Data

Policy information about availability of data

All manuscripts must include a data availability statement. This statement should provide the following information, where applicable:

- Accession codes, unique identifiers, or web links for publicly available datasets
- A description of any restrictions on data availability
- For clinical datasets or third party data, please ensure that the statement adheres to our policy

The data supporting the findings reported in this paper are included in the article and extended data. Source data for all relevant figures are included with the article. The structure factor file and associated atomic coordinates have been deposited in the Protein Data Bank, accession code 9EJW.
Data relating to the structure of murine CD44 (PDB code 2JCP) and m04 (PDB code 4PN6) are accessible from the Protein Data Bank (https://www.rcsb.org/)
The m11 sequence is available from GenBank (https://www.ncbi.nlm.nih.gov/) accession code: CAP08055.1
Requests for materials can be directed to M. Degli-Esposti.

Research involving human participants, their data, or biological material

Policy information about studies with human participants or human data. See also policy information about sex, gender (identity/presentation), and sexual orientation and race, ethnicity and racism.

Reporting on sex and gender	N/A
Reporting on race, ethnicity, or other socially relevant groupings	N/A
Population characteristics	N/A
Recruitment	N/A
Ethics oversight	N/A

Note that full information on the approval of the study protocol must also be provided in the manuscript.

Field-specific reporting

Please select the one below that is the best fit for your research. If you are not sure, read the appropriate sections before making your selection.

☒ Life sciences ☐ Behavioural & social sciences ☐ Ecological, evolutionary & environmental sciences

For a reference copy of the document with all sections, see nature.com/documents/nr-reporting-summary-flat.pdf

Life sciences study design

All studies must disclose on these points even when the disclosure is negative.

Sample size	Group sample sizes were chosen based on standards in the field and our previous studies where we used power analysis to estimate group sizes that would provide at least 80% power to detect statistically significant differences.
Data exclusions	Exclusion criteria were pre-established i.e. data were only excluded if outliers were identified using the ROUT test.
Replication	All experiments were replicated at least twice, except for (i) Extended data Fig. 2 where infections were conducted in triplicate within the

Replication	same experiment and (ii) Fig. 5d and Extended data Fig. 7f where 56-97 white pulp areas were examined from 3 mice/group within one experiment. There were no failures of replication.
Randomization	Age- and sex-matched mice were randomly allocated to groups.<br><br>For in vitro experiments randomization was not required given there are no relevant covariates i.e. all samples were treated at the same time and analysed using the same equipment.
Blinding	Microscopy image analysis was performed in a blinded fashion by multiple investigators.<br>All other analyses were strictly quantitative; hence no blinding was required.

Reporting for specific materials, systems and methods

We require information from authors about some types of materials, experimental systems and methods used in many studies. Here, indicate whether each material, system or method listed is relevant to your study. If you are not sure if a list item applies to your research, read the appropriate section before selecting a response.

Materials & experimental systems

n/a	Involved in the study
☐	☒ Antibodies
☐	☒ Eukaryotic cell lines
☒	☐ Palaeontology and archaeology
☐	☒ Animals and other organisms
☒	☐ Clinical data
☒	☐ Dual use research of concern
☒	☐ Plants

Methods

n/a	Involved in the study
☒	☐ ChIP-seq
☐	☒ Flow cytometry
☒	☐ MRI-based neuroimaging

Antibodies

Antibodies used	Refer to tables "List of antibodies used for flow cytometry" and "List of antibodies used for immunofluorescence" provided in the manuscript.
Validation	All antibodies except for the anti-vCD44BP (M-627, 7G5), anti-IE1 (clone 6/58/1) and anti-CD11c (clone N148) are from commercial sources and validation is provided on the manufacturer's website.<br><br>The specificity of anti-vCD44BP antibodies was verified by comparing cells infected with virus expressing vCD44BP and a mutant lacking vCD44BP, referred to as ΔvCD44BP, or cells transfected with vCD44BP.<br><br>The anti-IE1 (clone 5/58/1) antibody was originally described in Koszonowski et al, J. Virol., 1987. Specificity was verified by the absence of staining of uninfected cells.<br><br>Anti-CD11c (clone N418) was provided by Meredith O'Keefe, Monash University, Australia and verified as described in doi: 10.1084/jem.20021031.<br><br>Antibody dilutions used in our experiments are reported in the Methods section, however they are only relevant to the specific batch used, and therefore investigators should perform their own titrations prior to use.

Eukaryotic cell lines

Policy information about cell lines and Sex and Gender in Research

Cell line source(s)	CV-1/EBNA<br>COS-7<br>EL4<br>IC-21<br>M2-10B4<br>SF9<br>Fibroblastic reticular cell line derived from murine C57BL/6 (FRC2)
Authentication	CV1/EBNA - sourced from ATCC (CCL-70)<br>COS-7 - sourced from ATCC (CRL-1651)<br>EL4 - sourced from ATCC (TIB-39)<br>IC-21- sourced from ATCC (TIB-186)<br>M2-10B4 sourced from ATCC (CRL-1972)<br>SF9 sourced from Thermo Fischer Scientific Cat# 11496-015<br><br>FRC2 was authenticated based on morphological assessment and flow cytometric analysis.

Mycoplasma contamination	The cell lines tested negative for mycoplasma.
Commonly misidentified lines (See ICLAC register)	No commonly misidentified cell lines were used.

Animals and other research organisms

Policy information about studies involving animals; ARRIVE guidelines recommended for reporting animal research, and Sex and Gender in Research

Laboratory animals	BALB/c, C57BL/6J and C57BL/6.CD45.1 were from the Animal Resources Centre, Ozgene ARC (Perth, Western Australia) or the Walter and Eliza Hall Institute of Medical Research (Melbourne, Victoria).<br>B6.Cd44-/- and B6.BALB-TC1 (TC1) (H2b NK1.1+ Ly49H-) were bred at Perkins Bioresources Facility (Perth, Western Australia).<br>BALB/c.Ifng-/- mice and BALB/c.Prf1-/- were obtained from the Animal Services Facility at QIMR Berghofer Medical Research Institute (Queensland, Australia).<br>Age-matched adult female mice (8-12 weeks old) were used for all experiments.<br><br>Mice were housed in Techniplast IVC Greenline cages. Room temperature 18-24 degrees Celsius with humidity 40-70% and a 12-hour light cycle (7am-7pm).
Wild animals	The study did not use wild mice.
Reporting on sex	Refer to Methods (Mice). Female mice were used.
Field-collected samples	No field collected samples were used in this study.
Ethics oversight	All animal experimentation was performed with ethics approval from Monash University Ethics Committee (MARP2); Perkins Animal Ethics Committees (for the Lions Eye Institute); University of Western Australia Animal Ethics Committee (for the Lions Eye Institute) and in accordance with NHMRC Australia Code of Practice for the Care and Use of Animals for Scientific Purposes.

Note that full information on the approval of the study protocol must also be provided in the manuscript.

Flow Cytometry

Plots

Confirm that:

☒ The axis labels state the marker and fluorochrome used (e.g. CD4-FITC).

☒ The axis scales are clearly visible. Include numbers along axes only for bottom left plot of group (a 'group' is an analysis of identical markers).

☒ All plots are contour plots with outliers or pseudocolor plots.

☒ A numerical value for number of cells or percentage (with statistics) is provided.

Methodology

Sample preparation	Refer to Methods: Isolation of leukocytes and fibroblastic reticular cells, cell staining and flow cytometric analysis
Instrument	BD FACSymphony A3 (Special Order Research Product), Serial Number: R66093723001
Software	BD FACSDiva Software, Version 9.1
Cell population abundance	Abundance of cell populations are indicated in the figures of the manuscript.
Gating strategy	The relevant gating strategies are presented in Extended data Figure 10.

☒ Tick this box to confirm that a figure exemplifying the gating strategy is provided in the Supplementary Information.
